# The Antihypertensive Drug Telmisartan Protects Oligodendrocytes from Cholesterol Accumulation and Promotes Differentiation by a PPAR-γ-Mediated Mechanism

**DOI:** 10.3390/ijms22179434

**Published:** 2021-08-30

**Authors:** Antonietta Bernardo, Mariagiovanna Malara, Lucia Bertuccini, Chiara De Nuccio, Sergio Visentin, Luisa Minghetti

**Affiliations:** 1 National Center for Research and Preclinical and Clinical Evaluation of Drugs, Istituto Superiore di Sanità, 00169 Rome, Italy; sergio.visentin@iss.it; 2 Institute for Anatomy and Cell Biology, Ulm University, 89081 Ulm, Germany; mariagiovanna.malara@uni-ulm.de; 3 Core Facilities, Istituto Superiore di Sanità, 00169 Rome, Italy; lucia.bertuccini@iss.it; 4 Research Coordination and Support Service, Istituto Superiore di Sanità, 00169 Rome, Italy; chiara.denuccio@iss.it (C.D.N.); luisa.minghetti@iss.it (L.M.)

**Keywords:** cholesterol accumulation, Niemann Pick type C disease, myelination, PPAR-γ, oligodendrocyte progenitors, drug repositioning

## Abstract

Our previous studies have demonstrated that specific peroxisome proliferator-activated receptor-γ (PPAR-γ) agonists play a fundamental role in oligodendrocyte progenitor (OP) differentiation, protecting them against oxidative and inflammatory damage. The antihypertensive drug Telmisartan (TLM) was shown to act as a PPAR-γ modulator. This study investigates the TLM effect on OP differentiation and validates its capability to restore damage in a pharmacological model of Niemann-Pick type C (NPC) disease through a PPAR-γ-mediated mechanism. For the first time in purified OPs, we demonstrate that TLM-induced PPAR-γ activation downregulates the type 1 angiotensin II receptor (AT1), the level of which naturally decreases during differentiation. Like other PPAR-γ agonists, we show that TLM promotes peroxisomal proliferation and promotes OP differentiation. Furthermore, TLM can offset the OP maturation arrest induced by a lysosomal cholesterol transport inhibitor (U18666A), which reproduces an NPC1-like phenotype. In the NPC1 model, TLM also reduces cholesterol accumulation within peroxisomal and lysosomal compartments and the contacts between lysosomes and peroxisomes, revealing that TLM can regulate intracellular cholesterol transport, crucial for myelin formation. Altogether, these data indicate a new potential use of TLM in hypomyelination pathologies such as NPC1, underlining the possible repositioning of the drug already used in other pathologies.

## 1. Introduction

Telmisartan (TLM) is an antihypertensive drug belonging to the angiotensin II receptor blockers (ARBs), commonly referred to as Sartans. These drugs are widely used to treat cardiovascular disorders, metabolic syndrome, and kidney disease [1]. It can reduce blood pressure and regulate glucose and lipid metabolism without causing obesity while decreasing triglyceride levels. In addition to acting as an ARB, TLM has a peculiar characteristic compared to other Sartans, being able to interact with the nuclear peroxisome proliferator-activated receptor-γ (PPAR-γ) and supports its activation with a specific conformational change and coregulatory recruitment profile compared to selective PPAR-γ agonists such as Thiazolidinediones [2]. This peculiar feature could help to minimize the side effects associated with Thiazolidinediones or other PPAR-γ agonists [3,4,5]. TLM is identified as a Selective Modulator of PPAR-γ activity (SPPAR-γM) and is a bifunctional ligand due to its ability to act, both as an angiotensin II receptor (AT1) antagonist and as a partial agonist of PPAR-γ [6,7,8].

PPAR-γ is a transcription factor belonging to the superfamily of nuclear receptors whose main activity is controlling target gene expression in fundamental processes, such as lipid metabolism, differentiation, and inflammation. Recently, PPAR-γ has attracted particular attention since it was proven helpful as a therapeutic target in a wide variety of brain disorders, including degenerative diseases, stroke, traumatic injury, and demyelinating diseases [9,10,11,12]. Therefore, identifying new agonists that can bind and act through this nuclear receptor without eliciting the side effects described for several of the known agonists is of great interest.

Until now, TLM activity at the cerebral level was poorly understood. However, recent studies have shown neuroprotective capabilities that are independent of the blockage of AT1 and have been attributed to the modulation of PPAR-γ. For example, TLM prevented apoptosis in neuronal cells exposed to nutrient deprivation and protected against neurotoxicity caused by high glutamate concentrations. TLM also attenuates inflammatory processes in astrocytes, microglia, and cerebrovascular endothelial cells [1,12], promotes neuroprotection in Parkinson’s animal models [13], is a promising drug in managing cognitive impairment and amyloidogenesis [14,15] and shows a protective effect in cuprizone-induced demyelination and behavioral dysfunction in mice [16]. Besides, it also acted as a potential neuroprotective agent preventing cognitive impairment in patients with vascular dementia [5,7,17,18].

Based on these neuroprotective actions and the notable role of PPAR-γ in promoting differentiation of oligodendrocytes (OLs) and the myelination process [19,20,21], we focused on the possible role of TLM in myelin-related diseases in the central nervous system.

The survival and correct differentiation of OL progenitors (OPs) are essential for the axon myelination process during development. Moreover, they are crucial for repairing the myelin sheath in demyelinating diseases such as Multiple Sclerosis [22,23] or diseases characterized by abnormal myelination, such as Niemann-Pick type C (NPC) disease, caused by mutations in one of the genes (*NPC1* or *NPC2*) encoding proteins involved in cholesterol transport. Therefore, the purpose of this paper was to verify whether TLM could promote OP differentiation and protect from damage, acting through the activation of the PPAR-γ.

## 2. Results

### 2.1. Effects of TLM on OP Cell Viability in Culture

**Figure 1:**
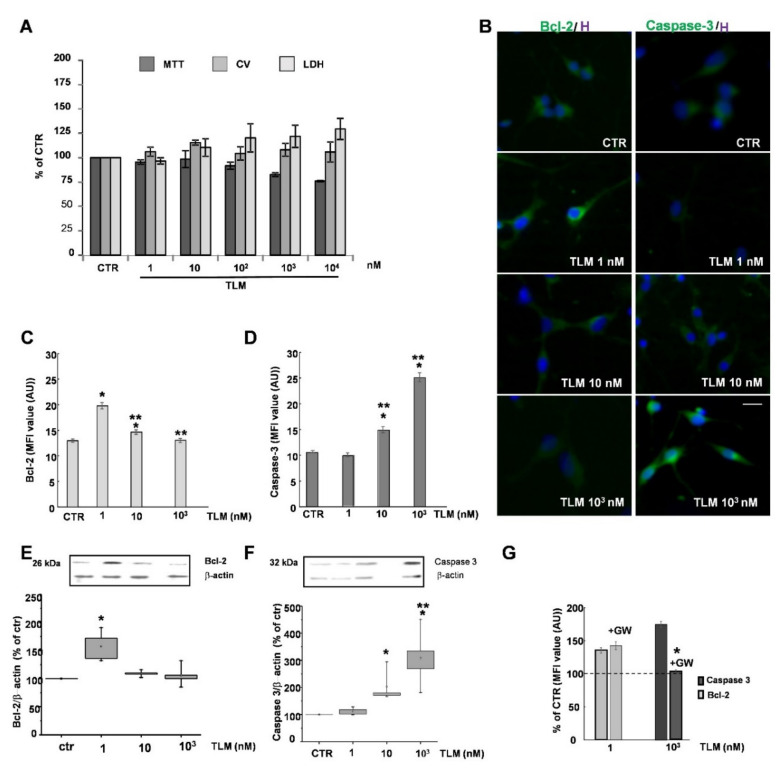
TLM modulates cell viability and anti- or proapoptotic cellular events. OP cultures were exposed for 24 h to different TLM concentrations (from 1 to 10^4^ nM). (**A**) Cell viability, total cell number, and cytotoxicity were evaluated by MTT, CV, and LDH assay. Means ± SEM are shown as percentage of control (*n* = 3–4; *p* = 6 × 10^−5^ for MTT and *p* = 0.0404 for LDH, one-way ANOVA). IF and WB techniques were used to assess BCL-2 (**B**,**C**,**E**) and Caspase-3 expression (**B**,**D**,**F**). Scale bar = 30 µm. The Means of fluorescence intensity (MFI) from 200–250 cells/condition/experiment are shown in (**C**,**D**). Representative WB of Bcl-2 or Caspase-3 and *β*-actin are shown (**E**,**F**). Band intensities are shown as % of CTR. *β*-actin is used as an internal control. Means ± SEM (*n* = 3; * *p* < 0.001 vs. CTR, ** *p* < 0.05 vs. 1 nM TLM, unpaired two-tailed Student *t*-test).The effect of PPAR-γ antagonist GW9662 (GW) is shown in (**G**) (*n* = 3, on 300–350 cells/experiment; * *p* < 0.005, unpaired two-tailed Student *t*-test).

To investigate TLM toxicity on highly purified OPs, we analyzed cell viability by MTT assay. Figure 1A shows that TLM 1 and 10 nM does not affect cell viability. In contrast, it decreases the capability of cells to reduce the tetrazolium salt in the range of 100 nM–10 μM. Moreover, at the same high concentration range, a significant increase in LDH release was observed, indicating membrane damage and possibly cell death. On the contrary, after 24 h of stimulation, the CV assay did not show alteration of the total cell number, with only a small but significant increase at low doses of TLM (Figure 1A). To better understand the suggested concentration-dependent toxicity of TLM, we analyzed the expression of antiapoptotic and proapoptotic proteins, Bcl-2 and Caspase-3. The levels of both proteins were assessed by immunofluorescence (IF) and Western Blot (WB) analysis. After 24 h of treatment with TLM, the expression of Bcl-2 was significantly increased in the cultures treated with the lowest concentration of TLM (1 nM: Figure 1B,C,E), whereas the levels of Caspase-3 increased in cells exposed to higher TLM concentrations (1 μM, Figure 1B,D,F). Pretreatment with GW9662, a specific PPAR-γ antagonist, in cultures stimulated with TLM concentrations capable of inducing the maximal expression of Bcl-2 (1 nM) or Caspase-3 (1 μM), blocked only Caspase-3 modulation, indicating that only the TLM toxic effects at high doses were mediated by the nuclear receptor (Figure 1G).

### 2.2. Telmisartan Activates PPAR-γ and Regulates AT1 Expression by a PPAR-γ-Mediated Mechanism

**Figure 2:**
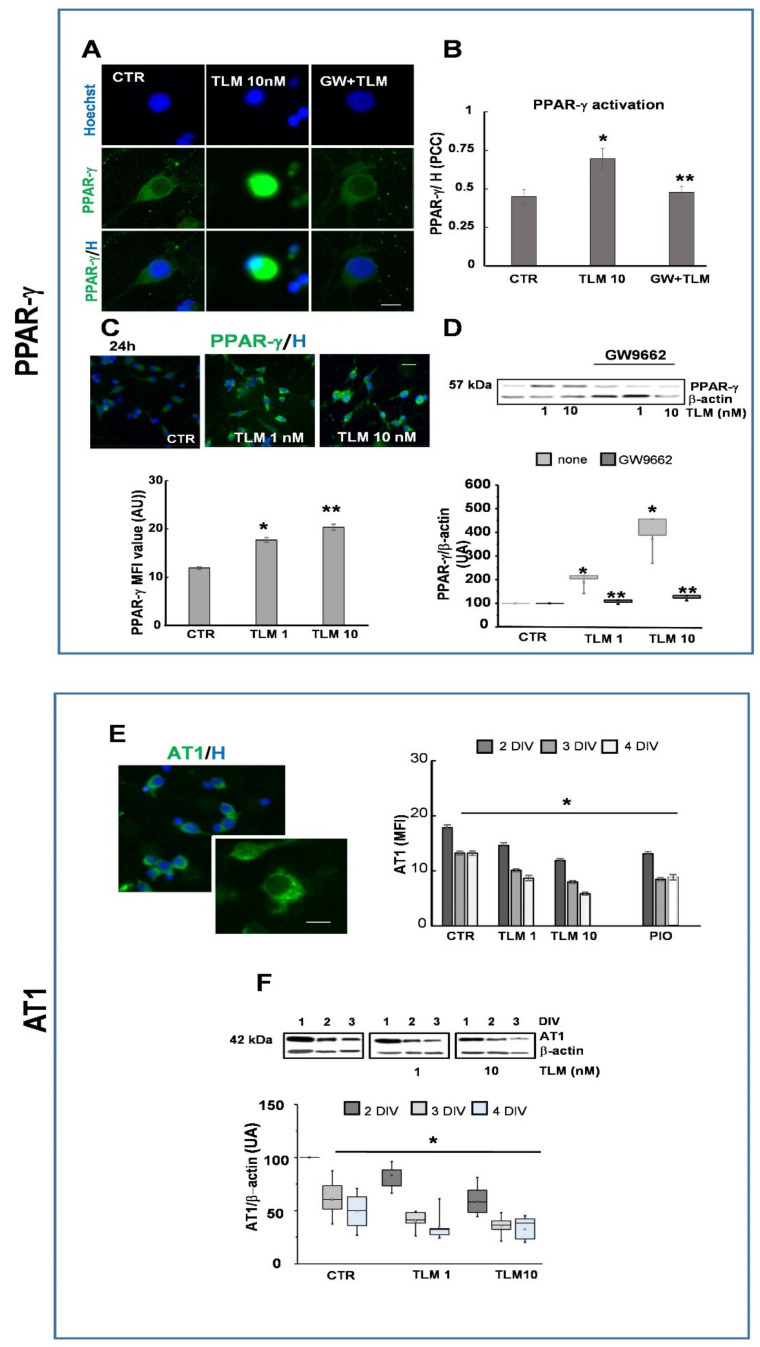
TLM increases the expression of PPAR-γ and modulates the AT1 receptor by a PPAR-γ mediated mechanism. OPs were treated for 60 min with 10 nM TLM in the presence or absence of the PPAR-γ antagonist GW9662 (GW) to study PPAR-γ activation. (**A**) Representative photomicrographs show PPAR-γ in green and nuclei in blue. The translocation of the receptor is shown as merging of PPAR-γ and Hoechst (H). Scale bar = 10 μm. Panel (**B**) shows the colocalization of the two signals (PPAR-γ/Hoechst nuclear labelling) expressed as the Pearson correlation coefficient (PCC). Data are shown as Means ± SEM *n* = 3; 300–400 cells (* *p* < 0.001 vs. CTR, ** *p* < 0.05 vs. TLM, unpaired two-tailed Student *t*-test). The PPAR-γ expression was evaluated by IF and WB analysis after 24 h of treatment with 1–10 nM TLM (**C**,**D**). Data for IF are shown as Means ± SEM; *n* = 3; 200–250 cells. Representative bands of PPAR-γ and β-actin are shown above, and densitometric analysis of 3 experiments is shown below (**D**) (* *p* < 0.001 vs. CTR, ** *p* < 0.05 vs. TLM, unpaired two-tailed Student *t*-test). OPs at 1DIV were treated with (1–10 nM) TLM and (1 μM) pioglitazone (PIO) for 24, 48, 72 h. The AT1 expression was evaluated at 2, 3 and 4DIV by IF and WB. (**E**) A representative photomicrograph and a higher magnification of a single cell is shown. AT1 expression is shown in green and nuclei in blue. Scale bar = 10 μm. The MFI from 200–250 cells/condition/experiment are shown (* *p* < 0.001 vs. untreated and treated at each DIV, unpaired two-tailed Student *t*-test, *p* = 0.0001 one-way ANOVA). (**F**) Representative bands of AT1 and β-actin are shown above, and densitometric analysis of 3 experiments is shown below (* *p* < 0.001 vs. untreated and treated at each DIV, unpaired two-tailed Student *t*-test, *p* = 0.00018 one-way ANOVA).

To verify whether TLM acts as a selective modulator of PPAR-γ in OP cultures, we first evaluated the activation of PPAR-γ. TLM induced the nuclear translocation of the PPAR-γ, an event that follows its interaction with the ligand and its activation. In control cultures, the receptor was visible at the cytosolic level. Conversely, after 60 min of treatment with 10 nM TLM the intense fluorescent signals were at the nuclear level, indicating its translocation. Such translocation/activation, shown as merging of PPAR-γ and Hoechst, was abolished by pretreatment with GW9662 (Figure 2A,B). Similar results were also obtained with 1 nM TLM (data not shown). Subsequently, we have verified the levels of PPAR-γ, after 24 h of treatment with (1–10 nM) TLM (Figure 2C,D). As expected for a PPAR-γ agonist, TLM increased receptor expression at both doses (evaluated by both IF and WB), and the presence of GW9662 abolished this effect (Figure 2D).

TLM is known as an antihypertensive drug that works by blocking the angiotensin receptor AT1. In the white matter in the rat adult CNS, this receptor is expressed in neurons and glial cells, including OLs [24]. However, the role of AT1 receptors in the physiology of the OL is entirely unknown. We first analyzed by IF the presence of the AT1 receptor in highly purified OP cultures. As shown in Figure 2E, at 2 days in vitro (DIV), the receptor is easily observable with a distinct dotted pattern (see detail in insert), whereas at 3DIV the expression tends to decrease, and to remain constant at 4DIV. Following treatment with 1–10 nM TLM, the level of AT1 is reduced as compared to control cultures at all time points studied (2, 3 and 4DIV; Figure 2E). WB analysis (Figure 2F) confirmed the observations.

To verify whether the modulation of AT1 could be related to the activation of PPAR-γ, as described in smooth muscle cells, we repeated the experiments using the specific PPAR-γ agonist pioglitazone (PIO, 1 μM), which significantly reduced AT1 expression over time, as for TLM (Figure 2E,F). Furthermore, the PPAR-γ antagonist GW9662, added to the cultures 30 min before TLM significantly reversed the effect of TLM on AT1 expression (MFI value: TLM 1 nM 14.2 + 0.65; TLM 1 nM + GW9662 16.1 + 0.7; TLM 10 nM 11.5 + 0.9; TLM 10 nM + GW9662 14.2 + 0.3; *p* < 0.05 vs. TLM, unpaired two-tailed Student *t*-test). Altogether, these results indicate that AT-1 expression is under the negative regulation of its agonist and PPAR-γ agonists.

### 2.3. Telmisartan Promotes the OP Differentiation in a PPAR-γ-Dependent Manner

**Figure 3:**
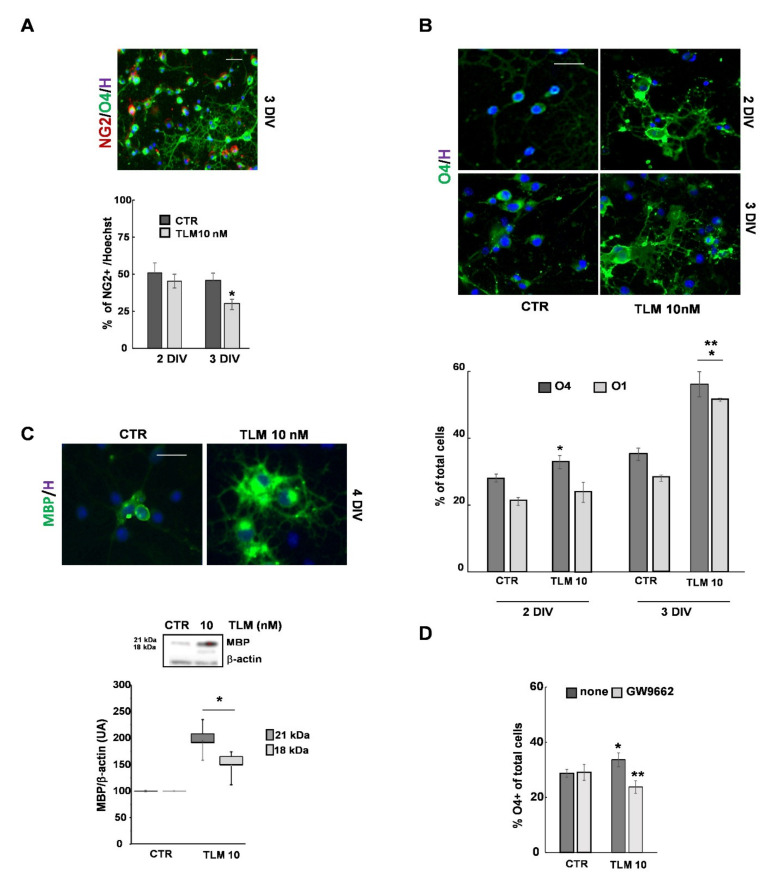
TLM promotes OP differentiation by a PPAR-γ mediated mechanism. At 1DIV, OPs were treated with 10 nM TLM for 24 or 48 h. The cells were processed for IF, and the expression of NG_2_, O_4_ and O_1_ was evaluated. Data are expressed as a percentage of positive cells to the marker with respect to the total cell number, highlighted by the nuclear dye Hoechst 33258. The photomicrograph in (**A**) shows examples of the morphology of cells at 3DIV, labelled with NG_2_ (red), O_4_ (green) and Hoechst (blue). Scale bar = 40 μm. The number of NG2 positive cells is evaluated in 3 independent experiments (* *p* < 0.001 vs. CTR, unpaired two-tailed Student *t*-test) in the graph below. The photomicrograph in (**B**) displays examples of the morphology of cells labelled with O_4_ (green) in control or TLM-treated cultures. The percentage of O_4_ and O_1_ positive cells are shown in the graph in (**B**). Data are presented as the Means ± SEM of 10 independent experiments (* *p* < 0.001 vs. CTR on the same experimental day, ** *p* < 0.05 vs. 2DIV, unpaired two-tailed Student *t*-test). (**C**) MBP expression was studied at 4DIV by IF and WB in OP cultures treated with TLM for 72 h. The anti-MBP antibody recognizes the two isoforms (18 and 21 kDa) of the protein in the WB assay. Data are the Means ± SEM of 3–4 independent experiments (* *p* < 0.001 vs. CTR, unpaired two-tailed Student *t*-test). Scale bar = 20 μm. (**D**) The percentage of O_4_^+^ cells is evaluated in the presence or absence of GW9662. Means ± SEM (*n* = 3–5; * *p* < 0.001 vs. CTR, ** *p* < 0.05 vs. TLM, unpaired two-tailed Student *t*-test).

Based on these initial analyses, we chose to use TLM at concentrations of 10 nM to study whether TLM, as previously demonstrated with other PPAR-γ agonists, could promote differentiation of OPs in mature OLs [20,21,25]. OP differentiation was assessed by evaluating specific markers related to distinct OL developmental stages. NG2 is expressed at both progenitor and pre-OL stages, O_4_ from the pre-OL stage onwards, O_1_ from the immature OL stage onwards; while the myelin basic protein (MBP) is expressed from the non-myelinating mature OL stage to the myelinating mature OL stage. At 2DIV, after 24 h in the presence of TLM, the percentage of NG2 positive cells was comparable to the control, while it was reduced after a further 24 h (3DIV) (Figure 3A). The percentage of O_4_^+^ cells was increased at both time points (2–3DIV), while O_1_^+^ cells were significantly increased at 3DIV only (Figure 3B). At 4DIV, the number of MBP positive cells, and MBP protein expression in the two isoforms of 21 and 18 kDa, were increased by TLM (Figure 3C). Moreover, OL differentiation induced by TLM was PPAR-γ-dependent, as suggested by the antagonistic effect of GW9662, at least in the initial phase characterized by O_4_ appearance (Figure 3D).

**Figure 4:**
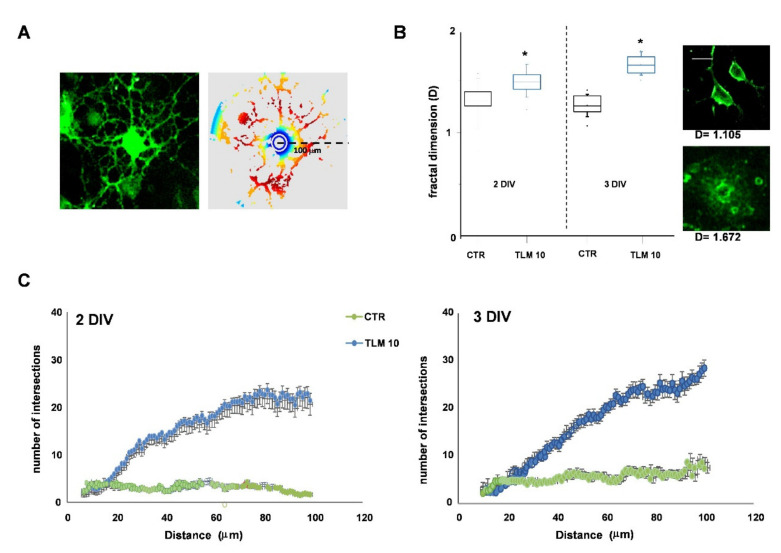
TLM increases the morphological complexity of OPs. Panel (**A**) shows a representative image of a single OP labelled with O_4_ and its corresponding schematic image reconstructed with ImageJ used for the Sholl analysis. At 1DIV, OPs were treated with 10 nM TLM for 24 or 48 h. The average number of intersections for each Sholl profile at 2–3DIV is shown in (**C**). Data represent Means + SEM (*n* = 3 independent experiments on 550–600 OPs; *p* < 0.001). The D values (fractal dimension) are shown in (**B**). Values represent at least 3 independent preparations on 50 OPs (* *p* < 0.0001 vs. CTR, unpaired two-tailed Student *t*-test). Representative images of cells studied are shown near the box plot graph. Scale bar = 20 μm.

It is known that the morphological complexity of OL processes is proportional to the differentiation state. To better quantify the complexity of such morphologies, we used two methods: fractal dimension analysis (D) [26,27] and Sholl analysis [28]. In the first case, lower D values are typical of cells with a progenitor phenotype with little branching of the processes. On the contrary, higher D values reflect a higher degree of morphological complexity of more differentiated cells. In the Sholl analysis, cells’ complexity is measured by evaluating the number of intersections that form in each cell, up to 100 µm from the cell body (Figure 4A). Although after 24 h of TLM treatment, the percentage of O_4_^+^ cells increased slightly, the cells showed a very pronounced morphological complexity (Figure 3B). This complexity was confirmed by both D values (Figure 4B) and the number of intersections formed (Figure 4C).

Finally, we analyzed the proliferating population after TLM treatment. We used the incorporation of bromodeoxyuridine (5-Bromo-2′-deoxyuridine—BrdU) together with the expression of the proliferating nuclear antigen (PCNA) [29] to evaluate the cells in the S phase. It is known that BrdU, an analogue of thymidine, is incorporated in proliferating cells during the S phase. On the contrary, PCNA is also expressed in the late G1 phase, and the onset of the S phase before DNA replication and its level decreases during the G2 phase and mitosis (Figure S1). To consider only S-phase cells (DNA synthesis) and not DNA repair [30], we measured the percentage of cells labelled simultaneously by PCNA and BrdU. In our conditions, we observed that at 2DIV, TLM induces coexpression of BrdU and PCNA comparable to the control cultures, whereas at 3 and 4DIV, TLM induces a decrease in both markers, consistent with TLM-induced differentiation (Graph in Figure S1).

### 2.4. Effects of Telmisartan in a Cellular Model of Niemann-Pick Type C Disease

Niemann-Pick type C (NPC) is a severe disease characterized in the CNS by dysmyelination and hypomyelination. The typical feature of the disease can be replicated by exposing cells in vitro to a pharmacological agent capable of inhibiting cholesterol transport, such as U18666A [31].

In a recent study [32], we exploited this model. We demonstrated that 1.25 µM U18666A induces in OP cultures several NPC1-like features such as accumulation of cholesterol, maturation arrest, altered mitochondrial morphology and function and defective autophagy, which was confirmed in this study by transmission electron microscopy data (Figure S2).

**Figure 5:**
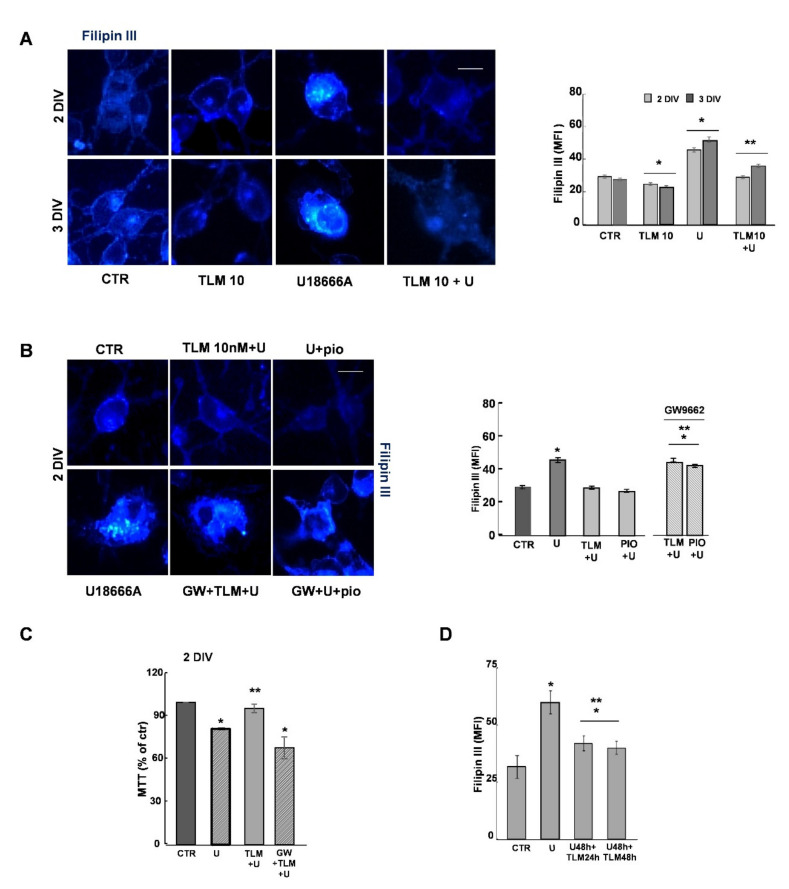
TLM redistributes the cholesterol in cultures treated with U18666A. (**A**) OPs were processed for Filipin III after a 24 h or 48 h treatment with 10 nM TLM and/or U18666A (U). Photomicrograph and the respective MFI values are shown. Data are expressed as Means ± SEM of 250–300 cells/condition (*n* = 3; * *p* < 0.001 and ** *p* = 0.048 vs. U18666A (one-way ANOVA, unpaired two-tailed Student *t*-test). Scale Bar = 10 μm. The OPs were treated with 1 μM GW9662 (GW) in the presence of TLM, and U18666A (TLM+U) or pioglitazone and U18666A (PIO+U), and the cholesterol distribution was evaluated in (**B**). Means ± SEM of 4 independent experiments are shown (250–300 cells/condition; * *p* < 7 × 10^−5^ vs. CTR, ** *p* < 8 × 10^−5^ vs. TLM+U or PIO+U; one-way ANOVA, unpaired two-tailed Student *t*-test). Scale Bar = 10 μm. The metabolic activity by the MTT test is shown in (**C**). Data are expressed as a percentage of control and represented the Means ± SEM of *n* = 5 * *p* = 0.001 and ** *p* = 0.039, one-way ANOVA, unpaired two-tailed Student *t*-test). The effect on cholesterol of TLM added in the last 24 h of U18666A is shown in (**D**). Data are Means of fluorescence intensity of Filipin III in each condition.

We tested TLM in these NPC1-like OLs, by treating OP cultures at 1DIV and evaluating the effects of the drug after 24 or 48 h (2 or 3DIV, respectively). As shown by Filipin III staining in Figure 5A, 10 nM TLM induced a redistribution of cholesterol when used alone or with U18666A. The effect of TLM was more pronounced at 3DIV when the accumulation of cholesterol and the morphological alterations induced by U18666A were more consistent (graph in Figure 5A). To demonstrate that the effect of TLM was dependent on PPAR-γ activation, we used the selective PPAR-γ agonist PIO, and the PPAR-γ antagonist GW9662. At the concentrations used, PIO and TLM had comparable effects, which were abolished by pretreatment with the PPAR-γ antagonist (Figure 5B). In addition, the PPAR-γ antagonist prevented the protective effect of TLM on metabolic activity, shown by the MTT assay (Figure 5C). Interestingly, the effect of TLM on cholesterol redistribution was also evident when the drug was added during the last 24 h of U18666A treatment, when the damage was already in place (Figure 5D).

**Figure 6:**
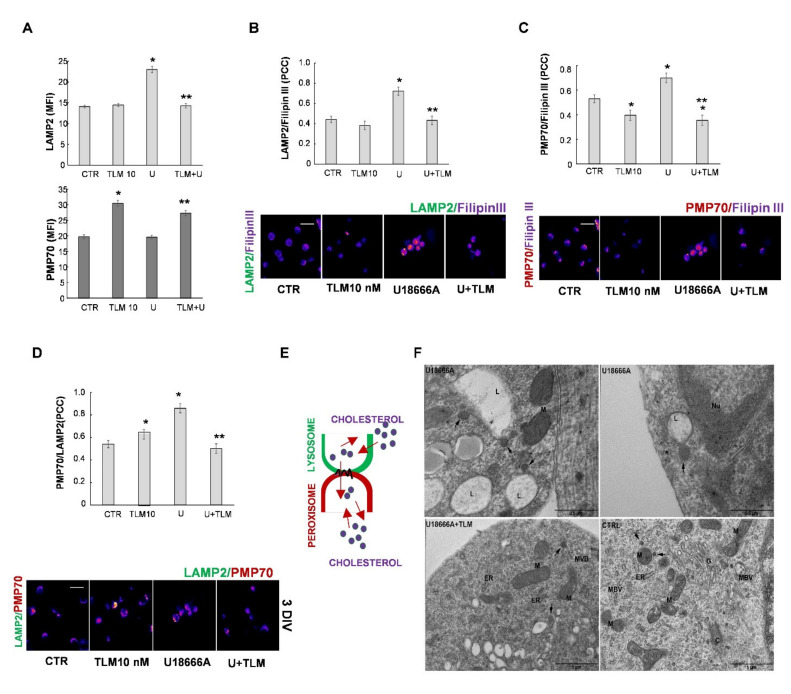
TLM modulates cholesterol traffic in lysosomes and peroxisomes. The lysosomal and peroxisomal levels were evaluated in OPs treated with TLM in the presence or absence of U18666A. The quantification of IF (MFI) of LAMP2 (lysosomal marker) and PMP70 (peroxisomal marker) is shown in (**A**). Data are Means ± SEM of 3 independent experiments (200–250 cells/condition; * *p* < 0.001 vs. CTR; ** *p* < 0.05 vs. U18666A, one-way ANOVA, unpaired two-tailed Student *t*-test). Colocalization of LAMP2/Filipin III (**B**) or PMP70/Filipin III (**C**) and LAMP2/PMP70 (**D**) were studied after 48 h of treatment with TLM 10 nM and/or U18666A (U). The photomicrographs evidence the localization of fluorescence signals captured and processed using ImageJ software, which reproduces the images according to a scale of arbitrary colors where the signal strength is proportional to the colocalization. Data are Means ± SEM of *n* = 3 (250–300 cells/field/condition). Scale Bar = 20 μm. The bar graphs indicate the quantification of several colocalizations, expressed as the Pearson correlation coefficient (PCC). In (**E**), The cholesterol traffic (red arrow) between a peroxisome and lysosome is shown in a schematic diagram. (**F**) TEM micrographs of OP treated for 48 h with U18666A, U18666A and TLM or untreated (CTR) are shown. The images show lysosomes (L) or peroxisomes (arrows) in close contact after U18666A treatment. M = Mitochondrion; ER = Endoplasmic Reticulum; G = Golgi apparatus; C = Centriole; Nu = Nucleus; MVB = Multivesicular Body. Scale Bar = 0.5–1 μm.

It is known that cholesterol is dynamically transported between different cellular compartments responsible for its synthesis, storage, and clearance. In addition to the autophagy mentioned above, where lysosomes and phagosomes/endosomes are involved, some other organelles, such as peroxisomes, play a critical role in the intracellular transport of cholesterol by establishing contact with lysosomes [33]. It is known that defects in peroxisomes and lysosomes contribute to the cholesterol accumulation characteristic of the NPC1 phenotype [34,35,36,37]. To study if TLM could affect the activities of these two organelles, we analyzed the possible modulation of levels of LAMP2 and PMP70, typical markers for lysosomes and peroxisomes, respectively. As shown in Figure 6A, TLM prevented the increase in LAMP2 levels induced by U18666A and increased PMP70 levels, also in the presence of U18666A.

Subsequently, we verified the accumulation of cholesterol in lysosomes and peroxisomes and the contact between these two organelles (Figure 6E). A 48 h treatment with 10 nM TLM reduced colocalization of Filipin III with the markers for both organelles (Figure 6B,C). The reduction in the colocalization between Filipin III and PMP70 in TLM treated cultures (Figure 6C) could be due to increased peroxisome levels suggested by the higher expression of PMP70. In addition, as shown in Figure 6D, U18666A increased the colocalization between LAMP2 and PMP70, and TLM reverted the effect to control levels. The TEM analysis of cells treated with U18666A showed a high frequency of contacts (Figure 6F upper panels), which was restored to the control condition by the TLM treatment (Figure 6F lower panels).

To assess OL differentiation, the percentage of O_4_^+^ and O_1_^+^ cells after 24 and 48 h (2–3DIV) of treatment with U18666A alone or in the presence of 10 nM TLM were analyzed. Table 1 shows the strong effect of TLM on OP maturation also in cultures exposed to U18666A. Of note, TLM was more effective in promoting differentiation in U18666A-treated cultures, as indicated by the highest percentage of O_4_^+^ and O_1_^+^ cells counted in this experimental condition at both time points.

**Figure 7:**
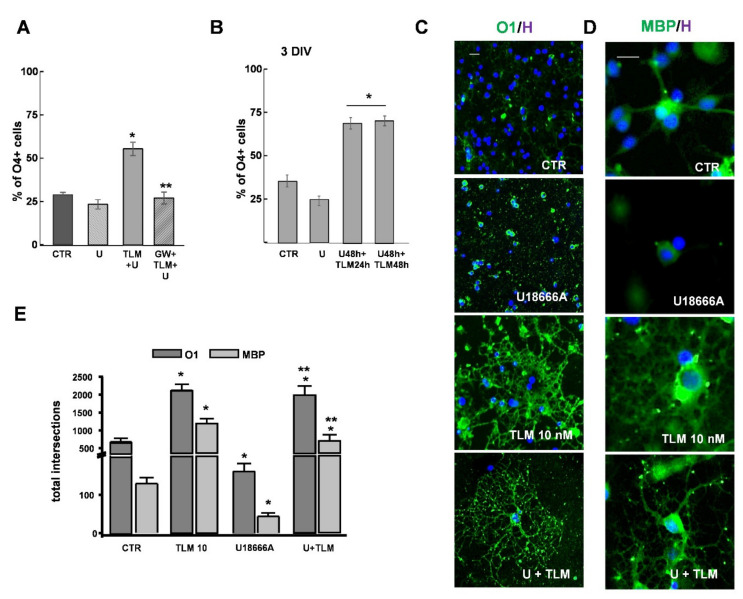
TLM reduces the damage induced by U18666A and promotes OP maturation. OPs were treated for 24 h with TLM 10 nM in the absence or presence of GW9662, and the percentage of O_4_ positive cells were evaluated on the total cell number in each condition (**A**). The data are the Means ± SEM of *n* = 4 (* *p* < 0.05 vs. U18666A; ** *p* < 0.0001 vs. TLM+U18666, one-way ANOVA, unpaired two-tailed Student *t*-test). The effect of TLM added in the last 24 h of U18666A on differentiation is shown (* *p* < 0.001 unpaired two-tailed Student *t*-test) (**B**). Data are the percentage of O_4_ positive cells evaluated on the total cell number in each condition. Cells after 48 h treatment with U18666A (U) and/or TLM at 10 nM (3DIV) are labelled with O_1_ (**C**) or anti-MBP (**D**) antibodies (green) and Hoechst 33258 (blue). The photomicrographs show the different cell morphologies—Scale Bar = 30 μm. The complexity of a single cell was assessed by Sholl analysis. The bar graph shows the presence of the total intersections for each Sholl profile delineated in the same cell (**E**). Data represent Means ± SEM *n* = 3 (50–60 cells/condition labelled for O_1_ or MBP; * *p* < 0.001 vs. CTR; ** *p* < 0.05 vs. U18666A, one-way ANOVA, unpaired two-tailed Student *t*-test).

We also tested TLM in U18666A treated cultures in the presence of the specific antagonist GW9662. As already shown in physiological conditions (Figure 3), the antagonist prevented the increase in the percentage of O_4_^+^ cells induced by TLM (Figure 7A). As before, TLM was effective even when added during the last 24 h of U18666A when the damage was already in place (Figure 7B).

Finally, the effect of TLM was also evident at the morphological level, as shown in Figure 7C–E, showing cultures exposed to U18666A and TLM labelled at 3DIV with O_1_ (C) or anti-MBP (D). Sholl analysis, considering the total number of intersections of a single cell, confirmed the decrease in cellular complexity in U18666A treated cultures and the increase in the presence of 10 nM TLM (Figure 7E).

Altogether, these observations indicate that TLM promotes functional recovery and differentiation of OPs exposed to U18666A by a PPAR-γ-dependent mechanism.

## 3. Discussion

This work aimed to evaluate the possible use of the antihypertensive drug TLM to promote myelination and thus, propose its potential use for therapeutic purposes in disorders affecting myelin. We used purified OL cultures capable of replicating differentiation in vitro, starting from OPs to mature OLs, and demonstrated that TLM promotes OP differentiation in control cultures and cultures exposed to a cholesterol transport inhibitor used as a pharmacological model of NPC1 OLs. Furthermore, we showed that these TLM effects are dependent on PPAR-γ-mediated mechanisms. 

In a series of previous studies, we showed that PPAR-γ is constitutively expressed in OLs from the most immature stages of the lineage and that both natural (15d-PGJ2, DHA, curcumin, phytoprostanes) and synthetic (pioglitazone) PPAR-γ agonists induce OP differentiation into mature OLs while increasing the intrinsic cellular mechanisms of defense against oxidative and inflammatory insults [19,20,21,25]. As far as the comparison of TLM and PIO, it is interesting to consider the different interactions of TLM and PIO with the PPAR-γ binding site. The former agent is classified as a partial agonist and the latter as a full agonist. Besides potentially causing the observed different kinetics of the effect, this might confer to TLM less transactivation potential compared to classical full agonists, which, from a therapeutic perspective, would confer to TLM a benefit due to potentially lower side effects. However, though a low dose of TLM induced upregulation of the antiapoptotic protein BCL-2 at higher concentrations (above 10 nM), TLM shared with more classical PPAR-γ agonists a proapoptotic capability, as shown by caspase-3 upregulation.

TLM is known to induce the activation of PPAR-γ in different cellular systems [6,38,39,40,41,42] due to its structural characteristics that allow PPAR-γ activation [43]. Our results indicate that TLM activates PPAR-γ in primary cells of the OL lineage, promoting its nuclear translocation and increasing its expression, as known for other PPAR-γ agonists [44].

TLM is an antagonist of the AT1 receptor, the expression of which has been detected in the white matter of the adult rat brain and possibly in OLs [24]. The role of AT1 in OL biology is unknown, but, interestingly, we found AT1 expressed in cells of the OL lineage at early stages, i.e., OPs, to be then reduced during differentiation. Consistent with the ability of PPAR-γ agonists to induce OL differentiation, TLM and pioglitazone, accelerated the decrease of AT1 levels by a GW9662-dependent mechanism, thus suggesting the possible involvement of PPAR-γ activation.

This hypothesis suggests a possible functional interaction between the two receptors, as already described. TLM was previously shown to protect from glutamate-induced excitotoxicity by a dual mechanism involving not only AT1 receptor blockade, but also a PPAR-γ-mediated downregulation of AT1 receptors. Similarly, the downregulation of AT1 receptors by TLM-induced PPAR-γ activation in smooth muscle cells was shown to contribute to the inhibition of the renin-angiotensin system [5,6].

In addition to providing evidence in favor of TLM as an inducer of OL maturation, by showing its effects on markers associated with postmitotic commitment and the initiation of terminal differentiation of OLs as well as on OL morphology, our study shows that TLM can protect and restore OL function by PPAR-γ-mediated mechanisms in a pharmacological model of Niemann-Pick type C (NPC) disease, which exploits the ability of U18666A to inhibit cholesterol transport from the lysosomal compartment. This event reproduces the main characteristic of the pathology, which is the formation of lipid deposits at the level of lysosomes and late endosomes both in neurons and in glial cells [45]. The formation of these deposits is likely associated with the arrest of OL maturation and the abnormalities of the myelin sheath [32,46]. 

In our experimental conditions, U18666A caused cholesterol accumulation, probed by Filipin III [47,48], which became more evident with time in culture, together with more substantial morphological alterations. Lipid deposits are in intracellular structures, identifiable as lysosomal clusters, based on their immunopositivity for a LAMP2-directed antibody and by TEM [32,49]. TLM protected cells from both U18666A-induced cholesterol accumulation and the retardation of differentiation by promoting cholesterol redistribution within cellular compartments, like lysosomes and peroxisomes, and reducing the interaction between organelles typically observed in pathological conditions [33,50,51].

The recovery of cholesterol homeostasis was accompanied by a marked differentiation, as indicated by increases in the percentage of cells expressing O_4_, O_1_ and MBP, which were even higher than observed in control cultures. This observation could suggest that under stress conditions, cells may become more sensitive to differentiating stimuli. Similarly, other PPAR-γ agonists, such as PIO, DHA or curcumin, were more effective in promoting OP differentiation under inflammatory stress, induced by the inflammatory cytokine TNF-α [21,52,53]. In a general view, the capability to respond to stress by entering a differentiation status might be considered a general feature of cells in the early stages of their development, as OPs are. Different environmental stresses in directing the differentiation of pluripotent stem cells are part of “new generation” methodologies applied to regeneration medicine [54]. However, we are not aware of the molecular determinants of such events in the specific case of PPAR-γ-induced differentiation.

Restoration of cholesterol distribution and the promotion of OL differentiation induced by TLM, in both control and stress conditions, were mediated by mechanisms involving PPAR-γ activation, as demonstrated by experiments using the PPAR-γ antagonist GW9662, which prevented both effects, and were further supported by similar activities observed with the specific PPAR-γ agonist PIO.

## 4. Materials and Methods

### 4.1. Cell Cultures

Purified cultures of OPs were prepared from newborn Wistar rats as previously described [25], following the European Communities Council Directive N. 86/609/EEC and using procedures approved by the Ministry of Health (authorization number: 152/2016-PR). OPs growing on top of mixed glial monolayers were mechanically detached and seeded at the density of 6 × 10^4^ cells/cm^2^ into poly-L-lysine-coated 35-mm diameter plastic culture dishes, 96 well plates, or 10/12 mm wide glass coverslips (for IF experiments). At 2 h after plating, the culture medium (DMEM with high glucose, supplemented with 10% FCS) was replaced with a chemically defined serum-free medium. This consisted of DMEM/HAMF12 (4:1), supplemented with 5.6 mg/mL glucose, 5 µg/mL insulin, 100 µg/mL human transferrin, 100 µg/mL bovine serum albumin, 0.06 ng/mL progesterone, 40 ng/mL sodium selenite, 16 µg/mL putrescine, bFGF, PDGF (PeproTech EC, Ltd., London, UK), 50 U/mL penicillin, 50 µg/mL streptomycin, and 2 mM glutamine. After 24 h, the cells were exposed to Telmisartan (TLM), pioglitazone (PIO), U18666A and the specific PPAR-γ antagonist GW9662, as detailed in figure legends. Other chemicals were from Sigma-Aldrich Italia (Milan, Italy).

### 4.2. Cell Viability

The ability of cells to reduce 3-(4,5-dimethyl thiazol-2-y1)-2,5-diphenyl tetrazolium bromide (MTT) was assessed as an index of cellular metabolic activity and mitochondrial integrity, as previously described [25]. MTT (Sigma, Saint Louis, MO, USA) was added at a final concentration of 0.25 mg/mL during the last 4 h of incubation. The medium was then removed, and 100 µL DMSO was added to each well to dissolve the dark blue crystals. The plates were then read on a microplate reader, using a test wavelength of 570 nm and a reference wavelength of 630 nm. The cell number in each condition was estimated by crystal violet (CV, Sigma, Saint Louis, MO, USA) dye [55]. Besides, to evaluate the membrane integrity, the release into the culture supernatant of the cytosolic enzyme lactate dehydrogenase (LDH) was measured using a colorimetric cytotoxicity assay kit (Roche Diagnostics, Milan, Italy).

### 4.3. Immunofluorescence

When the cells were characterized for antigen expression using the monoclonal antibodies O_4_ and O_1_, fluorescein-conjugated goat anti-mouse IgM (1:200, Jackson ImmunoResearch Laboratories, Inc., West Grove, PA, USA) was used as a secondary antibody before fixation in 4% paraformaldehyde, as previously described [25]. For double immunostainings, after fixation and permeabilization with 0.2% Triton X-100 for 10 min at RT, the cells were preincubated with 3% BSA in 0.1% Triton X-100/PBS solution for 1 h at RT and then incubated overnight at 4 °C with rabbit polyclonal anti-PPAR-γ, anti-AT1, anti-Caspase3 (1:100, Santa Cruz Biotechnology Inc., Dallas, TX, USA), anti-NG_2_ (1:100, Abcam, Cambridge, UK) or monoclonal anti-MBP (1:100, Millipore, Milan, Italy) in the same preincubation solution. Otherwise, cells were preincubated with 10% Goat serum/0.25% Triton X-100 for antibodies against LAMP2, PMP70 and Bcl2 (1:100, Abcam, Cambridge, UK). After 2 h at RT and extensive washing, secondary antibodies Cy3R IgG or FITC IgG polyclonal or monoclonal goat antibodies were used (1:200, Jackson ImmunoResearch Laboratories, Inc., West Grove, PA, USA). 

For Filipin III, the cells were incubated with 250 μg/mL Filipin III (Sigma, Munich, Germany). For double staining, cells already processed with primary and secondary antibodies were treated with glycine (1.5 mg/mL, PBS) for 10 min (RT) and then exposed to Filipin III (in the dark) for 30 min.

Cells exposed to 5-Bromo-2′-deoxyuridine (BrdU, final concentration 10 μM) (Roche Molecular Biochemicals, Mannheim, Germany) for 16 h were fixed with paraformaldehyde and incubated with 4% goat serum containing 0.05% Tween 20 for 20 min. The polyclonal anti-PCNA antibody (1:100 Santa Cruz Biotechnology Inc., Dallas, TX, USA) was then applied for 2 h and was visualized with Cy3R IgG anti-mouse (1:200, Jackson ImmunoResearch Laboratories, Inc., West Grove, PA, USA). After washing in PBS, the cells were exposed to the monoclonal anti-BrdU antibody using detection kit II (Roche Molecular Biochemicals) for 1 h (1:100) and to goat anti-mouse IgG (1:200, Jackson ImmunoResearch Laboratories, Inc., West Grove, PA, USA). Nuclei were stained using Hoechst-33258 (5 µg/mL for 20 min, Sigma, Munich, Germany). Coverslips were mounted with Vectashield Mounting Medium (Vector Laboratories, Burlingame, CA, USA) and examined using a Leica DM4000B fluorescence microscope equipped with a DFC420C digital camera and Leica Application Suite Software (260RI) for image acquisition (Leica, Wetzlar, Germany). The cells (approximately 100 cells/microscopic field) were counted in 10 microscopic fields of 0.18 mm^2^ per coverslip, prepared in duplicate for each condition from at least 3 independent experiments. Alternatively, images were obtained from labelled cells by capturing at least 8–10 photographs. All cells were evaluated for their fluorescence intensity and other morphological parameters by the software ImageJ.

### 4.4. Image Analysis and Quantification

IF analyses were conducted using NIH ImageJ software (URL: https://imagej.nih.gov/ij/index.html (accessed on 9 July 2014)). We considered the mean threshold fluorescence intensity within a region of interest, delineated by a single-cell profile, to determine the different expression levels of specific markers. Colocalization of particular proteins was analyzed by Pearson’s correlation coefficient (PCC) [56]. OP morphology was evaluated using two techniques: Sholl analysis and Fractal dimension (D) analysis of OL, using a specific plugin for ImageJ (URL: https://imagej.nih.gov/ij/index.html (accessed on 13 February 2018)). By Sholl analysis, the program analyses the number of intersections made by the branching of oligodendrocyte processes by constructing concentric circles on every cell that it considers, a distance from the cell body estimated within 100 microns (URL: https://imagej.net/plugins/sholl-analysis (accessed on 13 February 2018), [57]. Data were reported as the mean number of intersections per radius valued in the distance from the cellular body until 100 microns away from the center of cells, or as the total number of intersections in an entirely considered space. A minimum of 50 randomly chosen cells per condition with at least one process >50 μm were analyzed. D analysis was applied to evaluate the morphological complexity of cells. A numerical value close to 1 for cells with low morphological complexity (essentially bipolar cells) and near 2 for those with high complexity (highly branched cells or with a bidimensional planar structure), [26] was applied. This procedure was performed for at least 40 cells per condition for each independent experiment.

### 4.5. Transmission Electron Microscopy (TEM)

Cell monolayers were fixed in 2.5% glutaraldehyde, 2% paraformaldehyde, 2 mM CaCl_2_ in 0.1 M sodium cacodylate buffer, pH 7.4, overnight at 4 °C, and processed according to Perry and Gilbert. Cells were washed in buffer and post-fixed with 1% OsO_4_ in 0.1 M sodium cacodylate buffer for 1 h at RT, treated with 1% tannic acid in 0.05 M cacodylate buffer for 30 min and rinsed in 1% sodium sulphate in 0.05 M cacodylate buffer for 10 min. Postfixed specimens were washed, dehydrated through a graded series of ethanol solutions (30–100% ethanol) and embedded in Agar 100 (Agar Scientific, Monterotondo, RM, Italy). Ultrathin sections, obtained by a UC6 ultramicrotome (Leica), were stained with uranyl acetate and Reynold’s lead citrate and examined at 100 kV by a Philips EM 208S Transmission Electron Microscope (FEI-Thermo Fisher) equipped with an acquisition system Megaview III SIS camera (Olympus-SIS, Milan, Italy).

### 4.6. Western Blot Analysis

Cell lysates were prepared as previously described [25]. Briefly, OPs were lysed in ice-cold RIPA buffer (50 mM Tris-HCl supplemented with 1% NP40, 0.1% SDS, 0.1% Sodium desoxycholate, 1X Protease Inhibitor Cocktail, Sigma, 10 mM NaF, 100 µM Na_3_VO_4_) and insoluble material was removed by centrifugation (5000× *g* at 4 °C, 10 min). The protein concentration was measured by BCA protein assay (Thermo, Pierce, MO, USA), and equal amounts of proteins (30 µg for lane) were separated by 4–12% SDS-PAGE (Novex, Life Technologies, Carlsbad, CA, USA) and transferred to PVDF membranes (BioRad, Milan, Italy). Membranes were blocked with 5% BSA in TBST and incubated with polyclonal anti-AT1 (1:500), anti-Caspase3 (1:100) or with monoclonal anti-MBP (1:600), anti-Bcl2 (1:500), anti-PPAR-γ (1:500) antibodies overnight at 4 °C. The housekeeping gene actin was recognized by mouse anti-β-actin antibody (Sigma Aldrich; 1:5000, 1 h at RT). Horseradish peroxidase-conjugated anti-rabbit or anti-mouse IgG (Thermo, Pierce 1:10,000, 1 h at RT) and enhanced chemiluminescence ECL reagents (Thermo, Pierce) were used as a detection system. Band detection and image capture were performed by ChemiDoc XRS Densitometer (Bio-Rad, Milan, Italy) and Image LabTM 4.0 software. Band quantification was performed by Image LabTM 4.0 software; intensities of bands corresponding to the protein analyzed were normalized over the corresponding β-actin bands.

### 4.7. Statistical Analysis

Data are expressed as Means ± SEM of (n) independent experiments (run in duplicate). Statistical significance was evaluated using Student’s *t*-test or a one or two-way ANOVA or Bonferroni correlation for multiple comparisons. The numbers of independent experiments are indicated in the figure legends; *p* < 0.05 was accepted as statistical significance. The experimental events and statistical analysis of the data presented in this study tracked the approaches and standards used mainly in “in vitro” studies. In each experiment, the causes of variability and any residues resulting from unexpected errors of the replicates were well controlled, as shown by the low SEM. In addition, the Shapiro Wilk test assessed the normality of distribution and was only applied in viability tests and IF experiments.

## 5. Conclusions

In this study, we propose the antihypertensive drug TLM as a putative new therapeutic approach for treating diseases characterized by myelin dysfunctions by virtue of its unique activity among the members of the Sartans family as a PPAR-γ selective modulator [58,59]. The study uses a “drug repositioning” approach to identify a new therapeutic indication for a drug already in clinical use. The advantage of using drugs already approved for human consumption is to shorten the time to transfer primary research results into clinical practice, since the damage/benefit profiles have previously been defined for these drugs.

**Figure 8:**
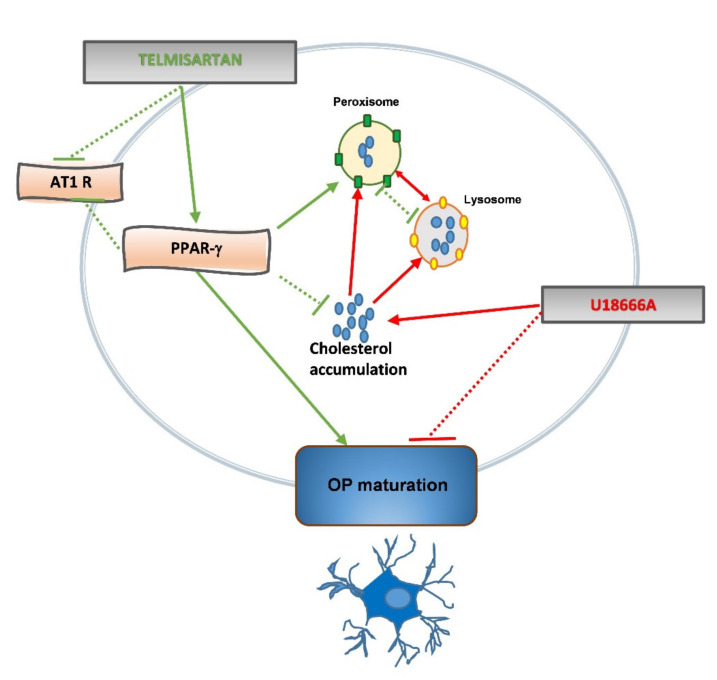
Schematic representation of TLM effects on OPs. TLM activates PPAR-γ in OPs, blocks AT1 activation, controls cholesterol metabolism and promotes OL differentiation by a PPAR-γ-dependent mechanism. TLM protects OP from the pathological effects induced by U18666A, modulating cholesterol traffic in the lysosome and peroxisome, and recovering maturation arrest. TLM and U18666A effects are illustrated by green and red lines, respectively. Solid arrows depict positive interactions. Broken lines indicate an inhibitory interaction.

The data obtained in our in vitro model (summarized in Figure 8) indicate that the antihypertensive drug TLM acts as an agonist of the PPAR-γ nuclear receptor in cells of OL lineage, promoting OP differentiation under physiological and pathological conditions, such as those mimicking the accumulation of intracellular cholesterol and morphological alterations typical of NPC disease. This study paves the way for further studies aimed at establishing the therapeutic potential of TLM in treating myelin diseases.

## Figures and Tables

**Table 1:** TLM rescues OP from maturational arrest induced by U18666A. The percentage of positive cells for O_4_ and O_1_ compared to the total cell number was evaluated after 24 h (2DIV) or 48 h (3DIV) of treatment in the presence of TLM and/or U18666A. The total cell numbers were counted using the vital nuclear dye Hoechst 33258. Data are Means + SEM of 5–10 independent experiments and evaluate 10–20 coverslips (* *p* < 0.02 vs. CTR ** *p* < 0.05 vs. U18666A; *** *p* < 0.0001 vs. TLM, one-way ANOVA, unpaired two-tailed Student *t*-test).

	% O_4_^+^ Cells		% O_1_^+^ Cells	
	2 DIV			
	none	U18666A	none	U18666A
CTR	28.7 ± 1	24.9 ± 2 *	22.4 ± 2	16.8 ± 2 *
TLM 10 nM	33.6 ± 3 *	58.3 ± 8 */**/***	24.7 ± 4	55.4 ± 4 */**/***
	3 DIV			
CTR	35.3 ± 3	27.1 ± 4 *	28.9 ± 2	22.7 ± 2 *
TLM 10 nM	56.4 ± 6 *	70.2 ± 4 */**/***	53.5 ± 1 *	68.4 ± 4 */**/***

## Data Availability

All data generated and analyzed are included in the published article, supplementary material and upon reasonable request to the corresponding author.

## Supplementary Materials

The following are available online at www.mdpi.com/article/10.3390/ijms22179434/s1, Figure S1. Effects of TLM on OP proliferation. Figure S2. TEM analysis of the impaired autophagy induced by the cholesterol transport inhibitor U18666A in OP cultures.

## Abbreviations

ARB	Angiotensin II receptor blocker
AT1	type 1 angiotensin II receptor
BrdU	5-Bromo-2′-deoxyuridine
CNS	Central Neurvus System
CTR	Control
CV	Crystal violet
D	Fractal dimension
DIV	Days in vitro
IF	Immunofluorescence
LAMP2	Lysosome-associated membrane protein 2
LDH	Lactate dehydrogenase
MBP	Myelin basic protein
MFI	Mean fluorescence intensity
MTT	3-(4,5-dimethyl thiazol-2-y1)-2,5-diphenyl tetrazolium bromide
NG2	Proteoglycan, Neural/glial antigen 2
NPC	Niemann-Pick type C
O1	galactocerebroside
O4	cell surface sulfatide
OL	Oligodendrocyte
OP	Oligodendrocyte progenitor
PCC	Pearson’s correlation coefficient
PCNA	proliferating nuclear antigen
PIO	Pioglitazone
PMP70	70-kDa peroxisomal integral membrane protein
PPAR-γ	Peroxisome proliferator-activated receptor gamma
RT	Room temperature
SPPAR-γ Ms	Selective Modulator of PPAR-γ activity
TEM	Transmission Electron Microscopy
TLM	Telmisartan
TNF-a	Tumor necrosis factor-alpha
WB	Western blot
