# Supplementary material for: The Antihypertensive Drug Telmisartan Protects Oligodendrocytes from Cholesterol Accumulation and Promotes Differentiation by a PPAR-γ-Mediated Mechanism
Source: Int J Mol Sci. 2021 Aug 30;22(17):9434. doi: 10.3390/ijms22179434 (PMC8431237; doi:10.3390/ijms22179434)
Supplement: Supplementary file 1 [file ijms-22-09434-s001.zip › ijms-1327779-supplementary.pdf]

The Antihypertensive Drug Telmisartan Protects Oligodendrocytes from Cholesterol Accumulation and Promotes Differentiation by a PPAR-γ-Mediated Mechanism

Antonietta Bernardo [1,*], Mariagiovanna Malara [2], Lucia Bertuccini [3], Chiara De Nuccio [4], Sergio Visentin [1] and Luisa Minghetti [4]

[1] National Center for Research and Preclinical and Clinical Evaluation of Drugs, Istituto Superiore di Sanità, 00169 Rome, Italy; sergio.visentin@iss.it
[2] Institute for Anatomy and Cell Biology, Ulm University, 89081 Ulm, Germany; mariagiovanna.malara@uni-ulm.de
[3] Core Facilities, Istituto Superiore di Sanità, 00169 Rome, Italy; lucia.bertuccini@iss.it
[4] Research Coordination and Support Service, Istituto Superiore di Sanità, 00169 Rome, Italy; chiara.denuccio@iss.it (C.D.N.); luisa.minghetti@iss.it (L.M.)

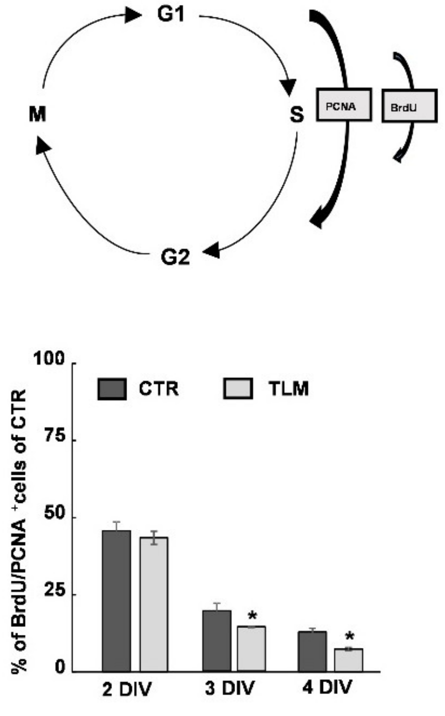

Figure S1. Effects of TLM on OP proliferation. The OPs were treated with TLM for 2, 3 or 4DIV and cell proliferation was evaluated. A schematic representation of the cell cycle phases with proliferation markers used is shown. The percentage of BrdU- and PCNA-positive cells compared to the total cells are shown below. The data represent the means±SEM of 3-4 experiments (* $p < 0.001$ vs CTR).

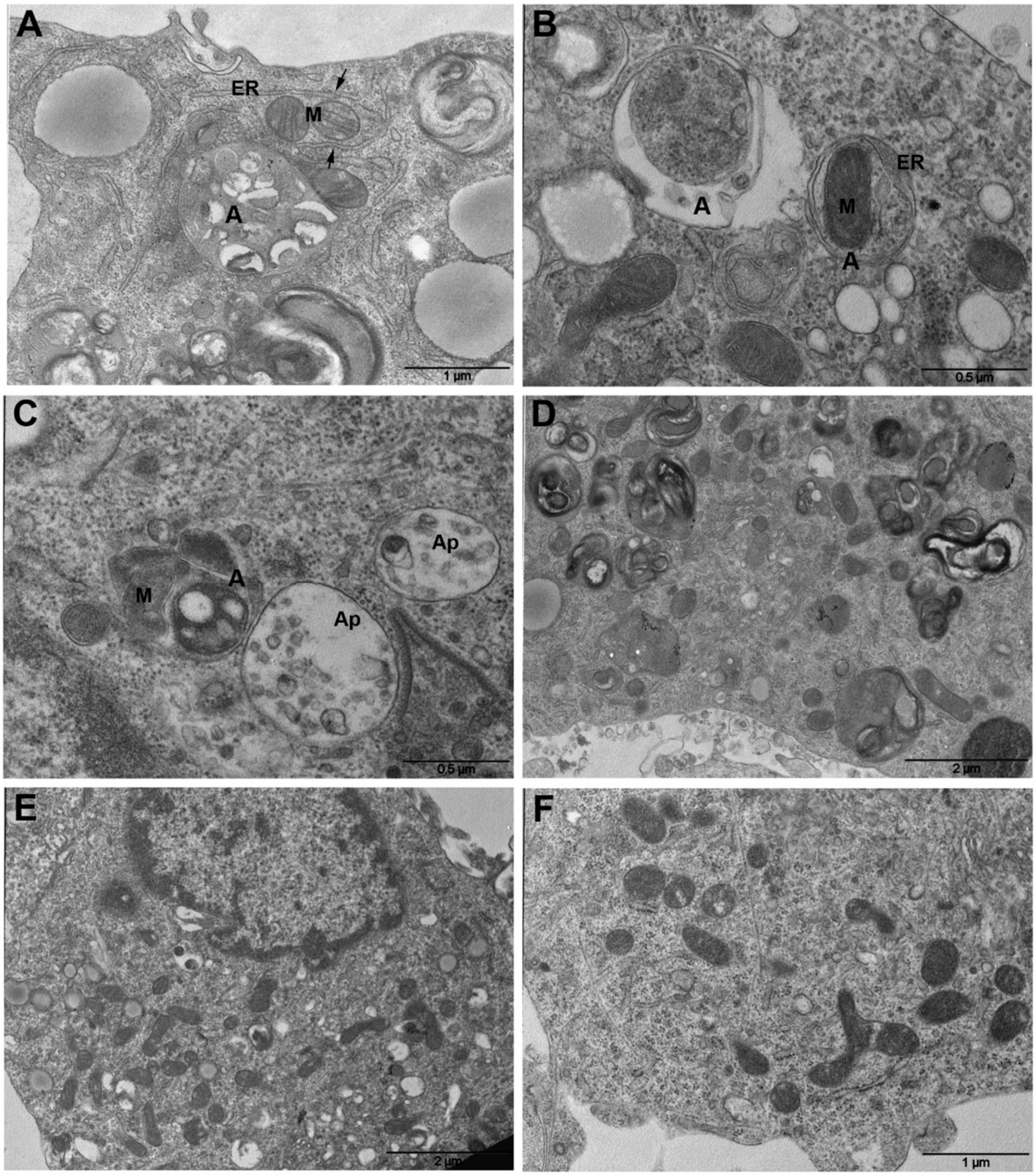

Figure S2. TEM analysis of the impaired autophagy induced by the cholesterol transport inhibitor U18666A in OP cultures. TEM micrographs of cells treated with U18666A are shown in A-D. In **panel A** the arrows indicate a phagophore (endoplasmic reticulum (ER) elongation around two mitochondria (M)). In **panel B** two newly formed autophagosomes (A) in which the cytoplasmic material and a mitochondrion are still intact are shown. Two amphisomes (Ap) and an autophagosome (A) are shown in **panel C**. In the micrograph the progression of digestion is evident, but the mitochondrion residue is still distinguished. In **panel D**, the altered autophagy is represented by many autolysosomes engulfing the cell cytoplasm. In **panel E**, TEM micrograph of an oligodendrocyte treated by U18666A and TLM is shown. The absence of autophagosomes engulfing the cell cytoplasm is evident. In **panel F** shows TEM micrograph of an untreated oligodendrocyte.
